# RSV Surge in the USA and Viral Lineages, 2022

**DOI:** 10.1056/NEJMc2216153

**Published:** 2023-02-22

**Authors:** Gordon Adams, Gage K. Moreno, Brittany A. Petros, Rockib Uddin, Zoe Levine, Ben Kotzen, Katelyn Messer, Sabrina T. Dobbins, Katherine C. DeRuff, Christine Loreth, Taylor Brock-Fisher, Stephen F. Schaffner, Sushma Chaluvadi, Sanjat Kanjilal, Jeremy Luban, Al Ozonoff, Daniel Park, Sarah Turbett, Katie J. Siddle, Bronwyn L. MacInnis, Pardis Sabeti, Jacob E. Lemieux

**Affiliations:** Massachusetts General Hospital, Boston, MA; Broad Institute of MIT and Harvard, Cambridge, MA; Broad Institute of MIT and Harvard, Cambridge, MA; Massachusetts General Hospital, Boston, MA; Broad Institute of MIT and Harvard, Cambridge, MA; Massachusetts General Hospital, Boston, MA; Broad Institute of MIT and Harvard, Cambridge, MA; Broad Institute of MIT and Harvard, Cambridge, MA; Broad Institute of MIT and Harvard, Cambridge, MA; Broad Institute of MIT and Harvard, Cambridge, MA; Broad Institute of MIT and Harvard, Cambridge, MA; Broad Institute of MIT and Harvard, Cambridge, MA; Broad Institute of MIT and Harvard, Cambridge, MA; Brigham and Women’s Hospital, Boston, MA; University of Massachusetts Chan Medical School, Worcester, MA; Broad Institute of MIT and Harvard, Cambridge, MA; Broad Institute of MIT and Harvard, Cambridge, MA; Massachusetts General Hospital, Boston, MA; Broad Institute of MIT and Harvard, Cambridge, MA; Broad Institute of MIT and Harvard, Cambridge, MA; Broad Institute of MIT and Harvard, Cambridge, MA; Massachusetts General Hospital, Boston, MA

## To the Editor:

The United States experienced an unusually early surge in respiratory syncytial virus (RSV) disease in autumn 2022 (Figure (Fig) 1A, 1B)^1^ for which the causes are unknown. To investigate whether the emergence of a highly transmissible or virulent variant contributed to the surge, we sequenced RSV genomes from a convenience sample of symptomatic patients diagnosed with RSV infection presenting to the Massachusetts General Hospital (MGH) in November 2022 Aand its outpatient practices in the Greater Boston Area. This work was approved by the Massachusetts General Brigham and MIT Institutional Review Boards. We believe this cohort was broadly representative, demographically and clinically, of national RSV patients (Supplemental (S) Tables 1A–C; Fig S1). Metagenomic whole genome sequencing of 105 residual diagnostic upper respiratory tract specimens produced 54 near-complete (>80% coverage) and 23 partial (>5% coverage) RSV genomes, primarily from samples with higher viral loads (Fig S2). These data also revealed viral respiratory co-infections with rhinovirus or enterovirus (9/105) or metapneumovirus (1/105; Fig S3).

**Figure 1:**
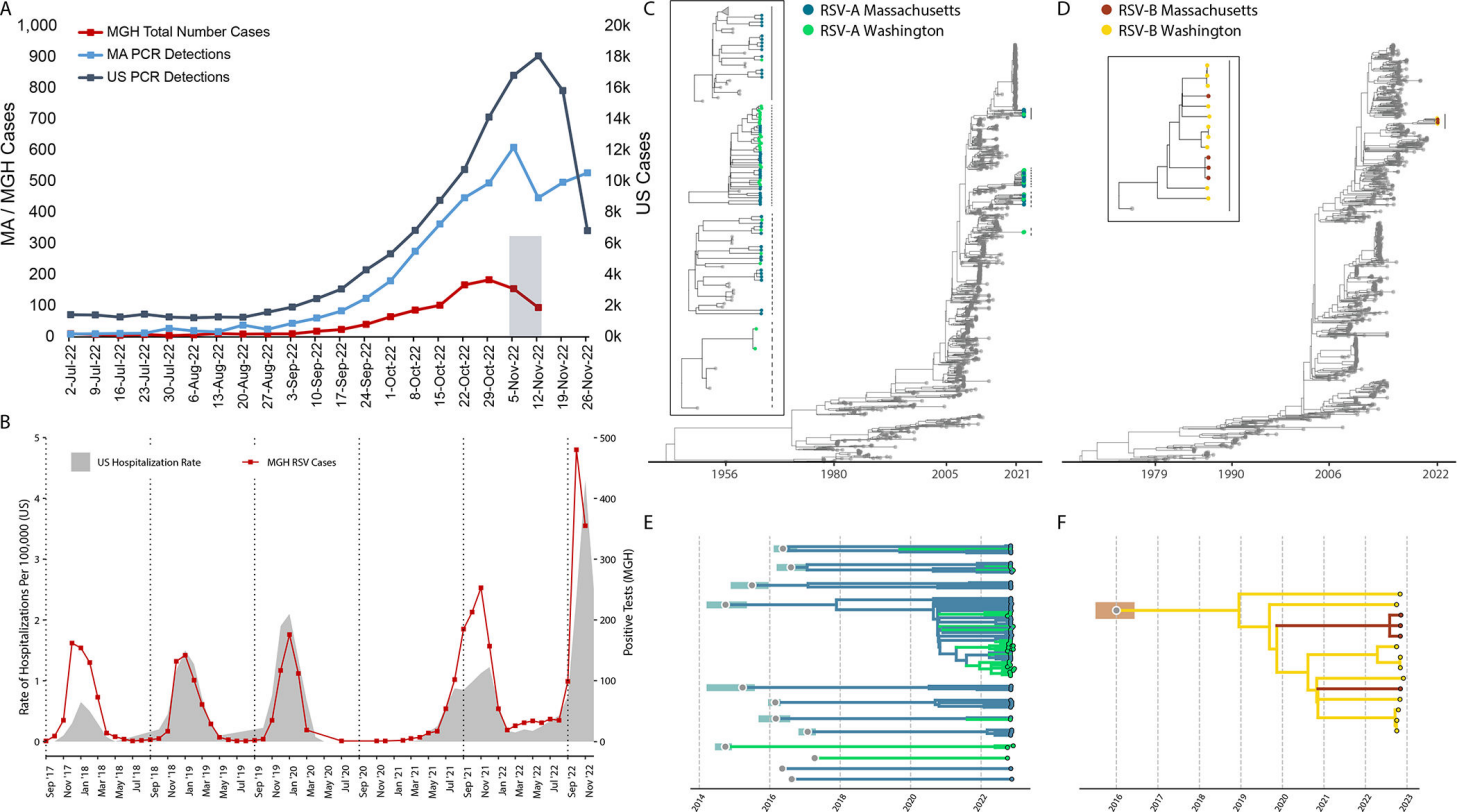
Epidemiological and genomic trends of the 2022 RSV surge. A) The number of PCR positive tests for RSV reported by the CDC in MA (blue, left axis) and the US (slate gray, right axis), and the number of RSV positive tests conducted at MGH (red, left axis). The 105 sequenced samples were drawn from the Nov 2 - Nov 15 window (shaded gray box). B) RSV hospitalization rates for CDC RSV-NET (shaded gray) and MGH RSV cases (red) for 2017 – 2022. (Pearson r = 0.82; p < 0.0001 via permutation). C) Maximum likelihood tree of all RSV-A genomes (N=1,267; MA genomes in blue, WA genomes in green, others in gray). The tMRCA for 2022 RSV-A genomes was no later than 2008 (ML CI: 2008–04, 2008–09). In the box are zoomed in plots of the clades containing MA and WA with lines corresponding to clades on the tree. D) Maximum likelihood tree of all RSV-B genomes (N=944; MA genomes in orange, WA genomes in yellow, others in gray). The tMCRCA for 2022 RSV-B genomes was approximately 2016 (ML CI: 2015–08, 2016–08). In the box are zoomed in plots of the clades containing MA and WA with lines corresponding to clades on the tree. Explosion plot of E) RSV-A and F) RSV-B lineages circulating in autumn 2022 with the inferred tMRCA (gray dots) and associated confidence intervals (shaded regions) for each lineage.

Genomic analysis demonstrated that the surge was driven by multiple lineages of RSV-A (91%; 70/77) and RSV-B (9%; 7/77) (Fig 1C,D; Fig S4A,B). All near-complete genomes were genotype GA2.3.5 (RSV-A) or GB5.0.5a (RSV-B). RSV-A genomes belonged to at least 10 distinct lineages, each with a time of their most recent common ancestor (tMRCA) between 2014 and 2017 (Fig 1C,E; Fig S4A; Fig S5A). The 4 complete RSV-B genomes similarly yielded a tMRCA estimate of 2019 as the later bound (Fig 1D,F, Fig S4B; Fig S5A). Currently, other publicly available RSV genomes from the 2022 surge in the US are from Washington^2^ (WA; N=39); the RSV-A genomes from WA belong to 6 lineages, of which 4 also contain MA genomes (Fig 1C,E; Fig S4A,B). The genetic divergence of the 2022 RSV-A and RSV-B genomes was consistent with our estimated clock rate from the larger phylogenetic tree (10.2 [9.8–10.7] and 10.7 [10.1–11.2] substitutions per year, respectively; Figure S5B,C), in contrast with the accelerated evolution seen with the highly transmissible SARS-CoV-2 variants^3,4^.

These data suggest the 2022 RSV surge in the US consisted of numerous pre-existing viral lineages, many shared between geographically disparate areas, and are inconsistent with the emergence of a single, highly transmissible RSV lineage as the cause of the surge. Non-viral factors—including changes in population immunity due to altered RSV dynamics, possibly resulting from interventions such as social distancing and masking during the COVID-19 pandemic—may impact the surge as well^5^.

## Supplementary Material

supplement
