# Supplementary material for: RSV Surge in the USA and Viral Lineages, 2022
Source: N Engl J Med. Author manuscript; Available in PMC 2023 Apr 7. (PMC10081154; doi:10.1056/NEJMc2216153)
Supplement: supplement [file NIHMS1875506-supplement-supplement.pdf]

Supplementary Appendix

Contents

Description of the Cohort……………………………………………………………….…….…….2 - 3

Supplemental Methods……………………………………………………………..……...……3 –9

Use of Data……………………………………………………………………………...………9

Ethical Statement………………………………………………………………………………9

Supplemental Figure S1…………………………………………………………….......…...10

Supplemental Figure S2………………………………………………………………….......11

Supplemental Figure S3………………………………………………………………….......12

Supplemental Figure S4…………………………………………………………………….…..13

Supplemental Figure S5…………………………………………………………….............14

Supplemental Table S1….…………………………………………………………..…..... 15 - 17

References……………………………………………..……………..………………………18 - 19

Description of the cohort

In autumn 2022, MGH reported a surge in RSV cases, with a total of 950 RSV-positive samples from Sept 1 – Nov 17 (**Supplemental Table 1**). In the MGH dataset, the number of positive RSV cases peaked the week of October 29, 2022, much earlier than previous years, and subsequently fell, mirroring trends that occurred at the state (**Figure 1A**), national (Pearson's correlation coefficient = 0.82, $p < 0.0001$; **Figure 1AB**)[1], and global levels[2].

The 77 patients from whom near-complete and partial RSV genomes were obtained consisted of 44% (n = 34) males with a median age of 2 years, and with 18% (n = 14) collected in inpatient care, 43% (n = 33) collected in outpatient care, and 39% (n = 30) collected in the emergency department (**Supplemental Table 1B**). All 77 patients reported symptoms consistent with a respiratory infection (**Supplemental Table 1B**). This cohort was representative of the greater MGH RSV-positive patient cohort with respect to multiple demographic criteria, including sex ($p = 0.12$, chi-square test), age ($p = 0.25$, Wilcoxon rank sum test), presenting hospital unit ($p = 0.07$, chi-square test), and presence of symptoms ($p = 1$, chi-square test) (**Supplemental Table 1A and B**). The MGH cohorts are also consistent with the national RSV hospitalization data[1] with respect to sex ($p > 0.05$, chi-square tests; **Supplemental Table 1C**) though they are slightly older than the national hospitalized cohort (median age 2 at MGH vs. < 1 nationally, $p < 0.0001$, **Supplemental Figure 1**). Our study (N = 77) was not powered to investigate associations between RSV genotype and clinical symptoms or outcomes (e.g., hospitalization or need for respiratory support). More broadly, the relationship between RSV subtype and clinical severity remains unclear[3-6].

We identified ten instances of co-infection with RSV and another known respiratory virus (9/105 rhinovirus or enterovirus, 1/105 metapneumovirus virus). We did not detect the presence of other common respiratory viruses (**Supplemental Figure 3**).

Genomic distances between samples ranged from 2 to 273 mutations for RSV-A, and 3 to 70 mutations for RSV-B, with a mean pairwise distance of 181 and 36 mutations respectively. We identified a single pair of near-complete genomes identical at the consensus-level, suggestive of a transmission link between these cases.

Methods

Description of samples and associated clinical data

For genomic analysis, we obtained 105 frozen (-80°C), archived, de-identified, residual diagnostic upper respiratory tract samples that were positive for RSV based on clinical diagnostic testing from the MGH Clinical Microbiology Laboratory, from individuals presenting with respiratory symptoms to MGH and its affiliated outpatient practices collected between November 2 – 15. Clinical diagnostic testing for the presence of viral respiratory pathogens was performed using one of three clinically verified, Food and Drug Administration (FDA) emergency use authorized or approved assays: the Xpert Xpress SARS-CoV-2/Flu/RSV (Cepheid, Sunnyvale, CA; either the original or plus assay; 90/105 samples) or the BioFire Respiratory 2.1 Panel (BioFire Diagnostics, Salt Lake City, UT; 15/105 samples). The Xpert Xpress SARS-CoV-2/FLU/RSV assays contain targets for the detection of SARS-CoV-2, influenza A and B, and RSV. The BioFire Respiratory 2.1 Panel contains targets for the following viruses: SARS-CoV-2, non-SARS-CoV-2 coronaviruses, human rhinovirus/enterovirus (combined target), influenza A and B, RSV, parainfluenza viruses 1-4, adenovirus, and human metapneumovirus.

For the full MGH RSV-positive cohort (N = 950), decisions regarding which assay was used for viral testing were based on MGH testing policy recommendations specific to the outpatient or emergency department (ED) and inpatient (IP) settings. For outpatients, from July 11, 2022 to October 18, 2022, testing using the Cepheid Xpress SARS-CoV-2/Flu/RSV or SARS-CoV-

2/Flu/RSV plus was recommended for symptomatic patients with risk factors for severe disease or complications from RSV (children < 3 years old, immunocompromised patients, or those with chronic lung disease). Routine testing for influenza was discouraged during this time due to low influenza prevalence based on surveillance data but was automatically performed (along with testing for SARS-CoV-2) when RSV testing was requested as these viral targets are included in the Cepheid assays. On October 18, 2022, based on guidance from the Centers for Disease Control and Prevention (CDC)[7] as well as an increase in influenza cases in Massachusetts, outpatient respiratory viral testing recommendations were changed to highlight appropriate indications for influenza testing, and the Cepheid assays were again used for symptomatic patients with risk factors for severe disease or complications from influenza[7]. RSV and SARS-CoV-2 testing were automatically performed when influenza testing was requested given the targets present in the Cepheid assays. The Cepheid assays were still the diagnostics of choice when RSV was clinically suspected. However, there was no requirement or verification that patients must meet these recommendations for testing. Expanded respiratory viral testing using the BioFire Respiratory 2.1 Panel was only available in the outpatient setting after approval by the MGH Clinical Microbiology Laboratory. For ED and IP testing, all respiratory viral testing was performed using the Pandemic Respiratory Order (PRO) set, which is a clinical decision support tool that uses provider responses to a series of predefined questions targeting clinical and operational indications for respiratory virus testing; the ordering clinician does not choose the assay used. Decision support rules were developed and maintained by experts in infectious diseases, infection control, and laboratory medicine based on national guidelines (e.g., the Infectious Diseases Society of America[8]) and operational considerations (e.g., discharge disposition), with input from other stakeholders. In general, testing using the Cepheid assays was performed on all adult immunocompetent patients presenting with symptoms consistent with COVID-19 as well as immunocompetent pediatric patients presenting to the ED with symptoms consistent with COVID-19 with a high likelihood of discharge home.

Immunocompromised patients (pediatric and adult) and immunocompetent pediatric patients presenting to the ED with symptoms consistent with COVID-19 with a high likelihood of hospital admission were tested using the BioFire Respiratory 2.1 Panel.

Patient demographics were extracted from the MGH Microbiology Sunquest Laboratory database, which collates patient information associated with all diagnostic samples collected at MGH. Symptom reports were extracted from the Mass General Brigham Enterprise Data Warehouse, which organizes Epic medical record data into an SQL database.

Processing and analysis of national data

Weekly 2022 national case counts were downloaded from the CDC[9], and weekly national hospitalization rates (with demographic breakdowns by age and sex) were downloaded from the RSV Hospitalization Surveillance Network (RSV-NET)[1]. We converted hospitalization rates per 100,000 individuals into case counts using demographics from the United Sates Census[10].

To compare monthly national case counts with MGH's monthly positive test counts, we calculated the Pearson correlation coefficient after removing months with missing data in either data set. To determine the significance of this correlation, the monthly national case counts were then scrambled across dates 10,000 times, generating a null distribution of correlation coefficients (**Figure 1B**).

The national hospitalization data binned ages (0-6 months, 6-12 months, 1-2 years, 2-5 years, 5-11 years, 12-17 years, 18-29 years, 30-39 years, 40-49 years, 50-64 years, 65-74 years, 75-84 years, 85+ years). For each case in each age bin, we randomly sampled an age uniformly across the bin. These sampled ages were compared to the ages of individuals in the MGH cohorts using the Wilcoxon rank sum test (**Supplemental Figure 1**).

Unbiased metagenomic sequencing

To prepare samples for sequencing, we extracted total nucleic acid from upper respiratory tract samples in transport media, removed DNA with DNase I treatment, and assessed viral quantity using an RSV-specific SYBR green RT-qPCR assay[11]. Human ribosomal RNA was then depleted with an RNase-H based method, and libraries were constructed using a strand-specific ligation-based approach[12]. Briefly, RNA was heat-fragmented and first-strand cDNA was generated with randomly primed reverse transcription. Second-strand cDNA was generated with nick translation and labeled with dUTP. Full-length, y-shaped, unique-dual-index Illumina sequencing adapters containing a UMI adjacent to the i7 index were ligated to the resulting double-stranded DNA. Samples were then treated with Uracil-Specific Excision Reagent (USER enzyme) to excise dUTP from the second strand. Libraries were PCR amplified, quantified, and pooled in equimolar ratios for sequencing on Illumina MiSeq or NextSeq 550 instruments.

RSV genome assembly and analysis

To assemble RSV genomes, we conducted all analyses using viral-ngs 2.1.28[13] on the Terra platform (app.terra.bio), available via the Dockstore Tool Registry Service (dockstore.org/organizations/BroadInstitute/collections/pgs). Briefly, samples were demultiplexed, reads were filtered for known sequencing contaminants, and de novo assembly with scaffolding against RSV-A (GenBank: KY654518.1) and RSV-B (GenBank: MZ516105.1) was performed.

The mean unambiguous genome length of these genomes was 11,970 bp for RSV-A and 10,268 bases for RSV-B. Assembled genomes with ≥50% completeness were deposited into NCBI GenBank (see Supplemental File 1). Raw reads for all samples (including those that did not produce a successful genome) were deposited in NCBI SRA. All NCBI data were deposited under BioProject PRJNA904288.

Phylogenetic analysis

We constructed RSV-A and RSV-B specific maximum-likelihood (ML) phylogenetic trees[14] with associated visualizations using an augur pipeline[15] (*augur_from_assemblies*), part of the Nextstrain project[16]. We included all contextual genomes from GenBank with reported collection dates and a genome length greater than 12,160 bp, corresponding to 80% of the reference genome length (resulting in a total of RSV-A N=1,238; RSV-B N=934 genomes downloaded on December 6, 2022). Within augur, RSV-A and RSV-B genomes were aligned via MAFFT v.7 to GenBank NC_038235.1 and NC_001781.1, respectively. This alignment was further processed in both the ML and Bayesian analyses.

We used the contextualized ML phylogeny to calculate the number of lineages circulating and estimate the time to tMRCA. To do so, we assigned a binary trait to each genome in the phylogeny, associated with the genome division of collection, and used Nextstrain's ancestral inference to infer the state of that trait for each internal node in the tree. We defined a lineage at the first node attributed to contain only MA descendants. Using baltic[17], we extracted these changes from the phylogenetic tree and plotted the inferred tMRCA for each lineage using matplotlib[18].

In parallel, we conducted molecular dating using BEAST version 1.10.5[19]. We used the same genome length filter (80% of the reference genome) and alignment described in the ML analysis. However, we used a subset of available genomes, selected as follows: (i) all genomes generated in 2022 (79 for RSV-A, 14 for RSV-B); (ii) all descendants of the parent node of all 2022 genomes (4 additional genomes for RSV-A, 0 additional genomes for RSV-B); and (iii) genomes sampled uniformly across time (221 additional genomes for RSV-A, 290 additional genomes for RSV-B), to reach a total of 304 genomes each for RSV-A and RSV-B. In BEAUti v.1.10.4, we defined two taxon sets for which we generated posterior distributions of the tMRCAs: one including all 2022

(i.e., MA and WA) genomes and one containing solely the 2022 MA genomes created in this study. We used dates with variable precision (i.e., retained sequences with missing day or month resolution) and used the HKY substitution model[20] with 4 categories of gamma site heterogeneity[21]. We combined a strict clock model with a coalescent tree prior (a piecewise Bayesian skyline model[22] with 5 groups). We ran BEAST twice, each with a UPGMA starting tree and 300 million MCMC steps, sampled every 1000 steps. We removed the first 10% of steps as burn-in and combined the two .log files via LogCombiner v1.10.4. We analyzed results in Tracer v.1.7.2[23] to ensure convergence, and isolated the molecular clock and tMRCA estimates from the combined .log file. We also combined the two trees files in LogCombiner v1.10.4, removing the first 10% as burn-in and sampling every 10,000 trees. We generated the maximum clade credibility (MCC) tree with median heights using TreeAnnotator v1.10.4[24] and displayed it using FigTree v1.4.4.

Using the molecular clock rate calculated by BEAST, we determined the residuals (number of mutations) per sample. Samples with a residual falling outside of the 2.5–97.5th percentiles were considered to statistically deviate from the molecular clock rate.

Root-to-tip plots were generated using TempEst[25].

Coinfection analysis

Among the RSV-positive sequenced samples, one co-infection was detected clinically via the BioFire Respiratory 2.1 Panel (RSV and rhinovirus/enterovirus). However, the majority of samples were not clinically assessed for most other co-infections, as the Cepheid assays only target SARS-CoV-2, influenza, and RSV. To assess for additional coinfections in all samples that underwent unbiased sequencing, taxonomic classification of reads was performed with Kraken2[26] using a custom database as previously described[27]. A viral taxon was considered present if >10 reads were assigned to it. We filtered the results to include solely known human respiratory

viruses. All Kraken2 classifications were verified by megablast as follows: first, megablast was run on *de novo* contigs. If the least common ancestor of the top e-value megablast hit agreed with the Kraken2 classification for at least one contig (≥200 bp), we considered the taxon present. If this criterion was not met, megablast was run on all reads assigned to the taxon of interest by Kraken2. If the least common ancestor of the top e-value megablast hits agreed with the Kraken2 classification for at least 90% of the assigned reads, the taxon was considered present

Use of data

Raw reads and assembled genomes are submitted to Genbank under PRJNA904288. The data are available immediately and shared under Genbank's use agreements to facilitate accelerated public health and scientific investigations. Files associated with the Bayesian analyses are available on GitHub at https://github.com/bpetros95/rsv-2022.

Ethical statement

This study was conducted at the Broad Institute and Massachusetts General Hospital with approval from the Massachusetts General Brigham Institutional Review Board under Protocol #2019P003305 and from the MIT Institutional Review Board under Protocol #1612793224. At MGH, clinical excess samples were collected from the Clinical Microbiology Laboratory, and associated clinical and demographic data were extracted from the electronic medical record and clinical laboratory information management systems under a waiver of consent.

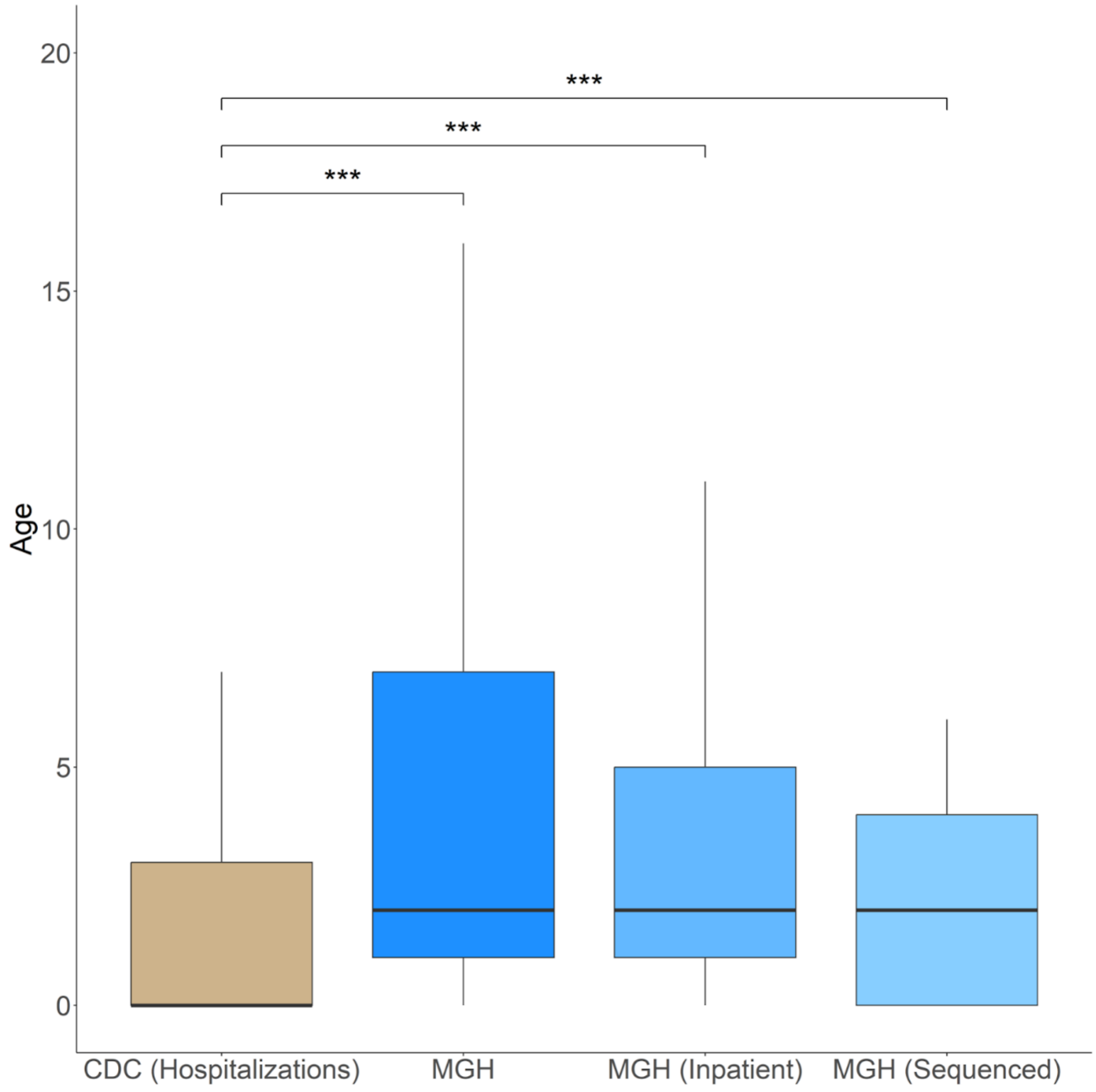

Supplemental Figure S1: Age of individuals in the national and MGH data sets. Median ages, < 1 year (CDC hospitalization data), 2 (MGH patients, N = 950), 2 (MGH hospitalized patients, N = 212), and 2 (MGH sequenced cohort, N = 77). ***, $p < 0.0001$ via Wilcoxon rank sum test.

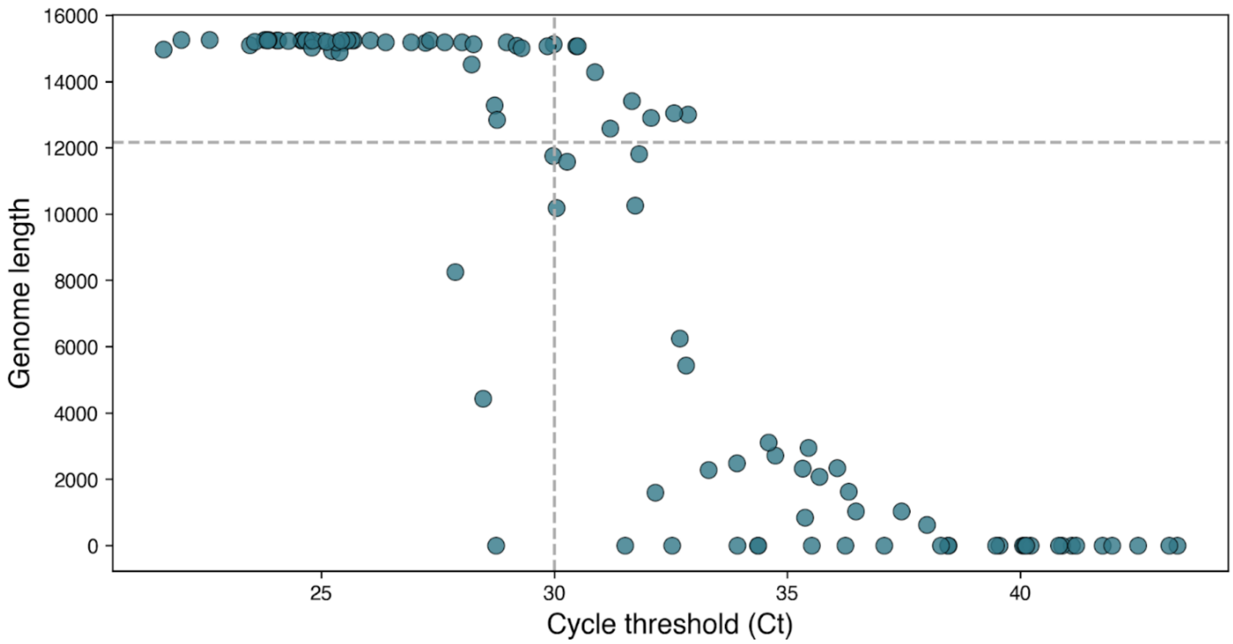

Supplemental Figure S2: Unambiguous genome length vs. cycle threshold (Ct). Each dot represents a unique sample in our dataset. Dashed lines are at a Ct of 30, and an unambiguous genome length of 12160, corresponding to ~80% of the RSV genome. Of the 105 samples, 92% (46/50) with a RT-qPCR Ct value ≤30 resulted in a genome, whereas only 15% (8/55) with a Ct >30 resulted in a genome.

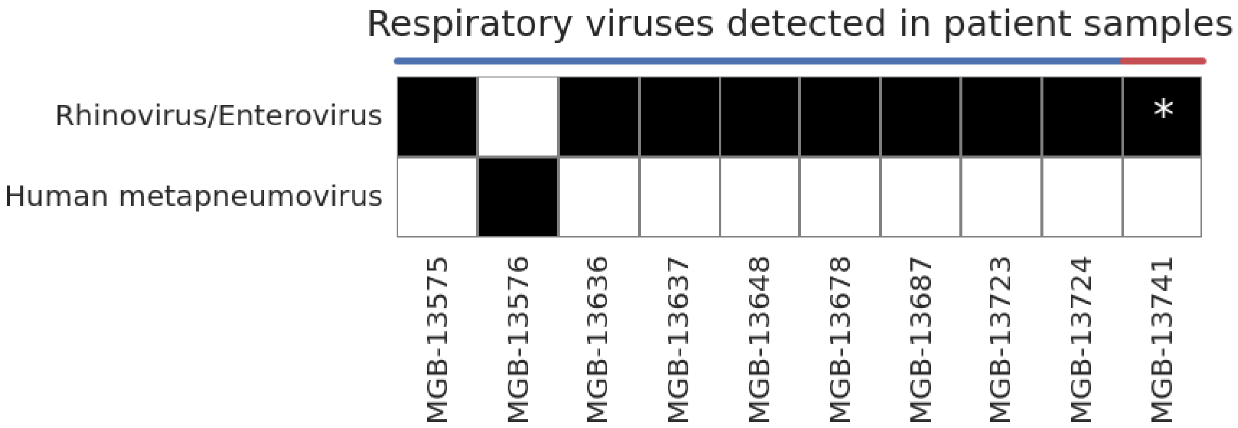

Supplemental Figure S3: Coinfection analysis of unbiased sequencing reads. Respiratory viral co-infections detected in patient samples by Kraken2 and megablast (Methods). Line indicates clinical testing with Xpert Xpress SARS-CoV-2/Flu/RSV (blue) or BioFire Respiratory 2.1 Panel (red). Star (*) indicates a positive clinical test.

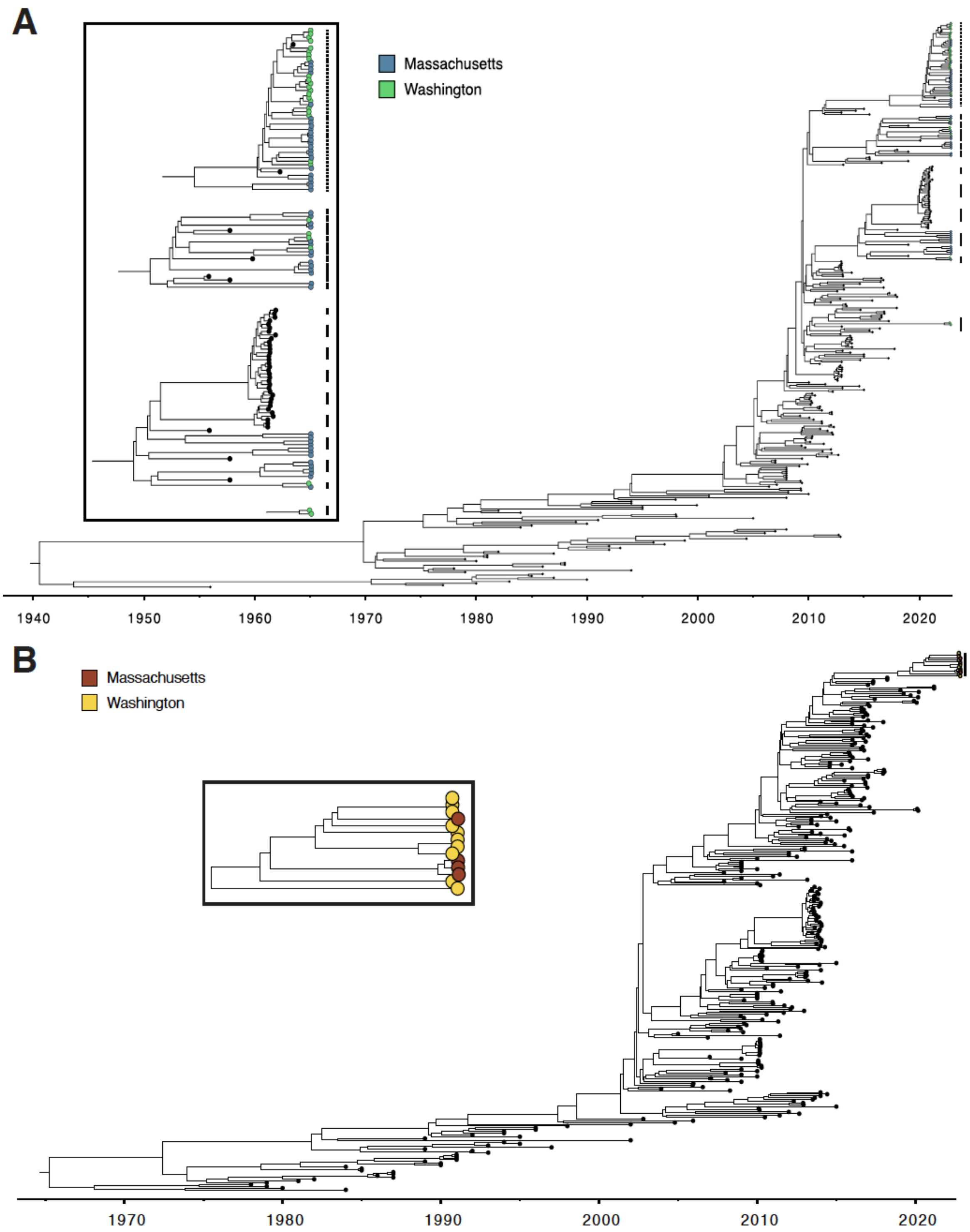

Supplemental Figure S4: Bayesian phylogenetic trees. A) Phylogenetic tree of a subset of RSV-A genomes (N=304; MA tips in blue, WA tips in green, others in black). The inset displays clades containing 2022 genomes with lines denoting their locations in the larger tree. B) Phylogenetic tree of a subset of RSV-B genomes (N=304; MA tips in red, WA tips in yellow, others in black). The inset displays the clade containing 2022 genomes with a line denoting its location in the larger tree.

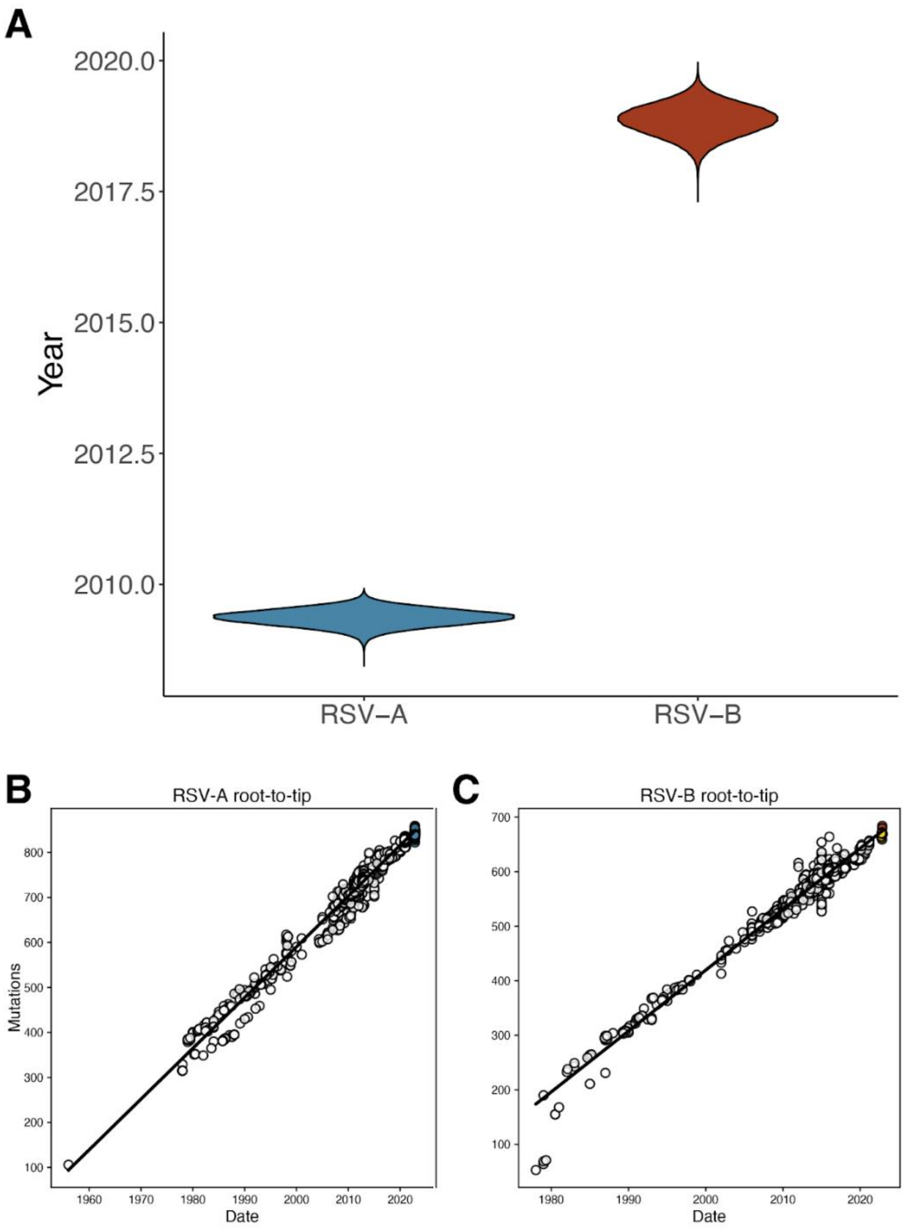

Supplemental Figure S5: Molecular dating of the 2022 RSV samples. A) Posterior distribution of the tMRCA for RSV-A (95% highest posterior density interval in blue, 2009-02 – 2009-08). Posterior distribution of the tMRCA for RSV-B (95% highest posterior density interval in red, 2018-03 – 2019-05). B) Root-to-tip mutation counts vs. sample collection date for RSV-A. MA tips in blue, WA tips in green. C) Root-to-tip mutation counts vs. sample collection date for RSV-B. MA tips in red, WA tips in yellow.

241 **Supplemental Table S1A. MGH sample demographics.**

	All Patients (N = 950)	Inpatient (N = 212)	Outpatient (N = 286)	Emergency Department (N = 452)
**Baseline Demographics**				
Median age (IQR) - yr	2 (0-5)	2 (1-5)	2 (0-6)	2 (0-4.3)
Male - no. (%)	507 (53.4)	100 (47.2)	162 (56.6)	244 (54.0)
**Reported Symptoms**				
Symptomatic - no. (%)	950 (100)	212 (100)	286 (100)	452 (100)
Cough - no. (%)	719 (75.7)	168 (79.2)	229 (80.1)	322 (71.2)
Fever - no. (%)	507 (53.4)	135 (63.7)	142 (49.7)	230 (50.9)
Sore Throat - no. (%)	90 (9.5)	21 (9.9)	23 (8.0)	46 (10.2)
Nasal Congestion/Runny Nose - no. (%)	361 (38.0)	92 (43.4)	149 (52.1)	120 (26.5)
Shortness of Breath - no. (%)	199 (20.9)	24 (11.3)	24 (8.4)	151 (33.4)
Loss of Smell or Taste - no. (%)	4 (0.4)	0 (0)	1 (0.3)	3 (0.7)
Muscle Aches - no. (%)	28 (2.9)	7 (3.3)	13 (4.5)	9 (2.0)

Supplemental Table S1B. Sequenced cohort demographics.

	All Patients (N = 77)	Inpatient (N = 14)	Outpatient (N = 33)	Emergency Department (N = 30)
**Baseline Demographics**				
Median age (IQR) - yr	2 (0-4)	1.5 (0.3-5.5)	1 (0-3)	2 (0.3-15.5)
Male - no. (%)	34 (44.2)	5 (35.7)	15 (45.5)	14 (46.7)
**Reported Symptoms**				
Symptomatic - no. (%)	77 (100)	14 (100)	33 (100)	30 (100)
Cough - no. (%)	62 (80.5)	9 (64.3)	28 (84.8)	25 (83.3)
Fever - no. (%)	43 (55.8)	9 (64.3)	17 (51.5)	17 (56.7)
Sore Throat - no. (%)	11 (14.3)	1 (7.1)	3 (9.1)	7 (23.3)
Nasal Congestion/Runny Nose - no. (%)	37 (48.1)	9 (64.3)	19 (57.6)	9 (30.0)
Shortness of Breath - no. (%)	15 (19.5)	0 (0)	4 (12.1)	11 (36.7)
Loss of Smell or Taste - no. (%)	0 (0)	0 (0)	0 (0)	0 (0)
Muscle Aches - no. (%)	3 (3.9)	0 (0)	2 (6.1)	1 (3.3)
**RSV Strain**				
RSV-A - no. (%)	70 (90.9)	13 (92.9)	28 (84.8)	29 (96.7)
RSV-B - no. (%)	7 (9.1)	1 (7.1)	5 (15.2)	1 (3.3)

Supplemental Table S1C. Number of cases (MGH and sequenced cohorts) or hospitalization rate per 100,000 (CDC) by sex. P-values calculated relative to the CDC data using the chi-square test.

	Female	Male	P-Value
CDC (Hospitalizations per 100,000)	24.8	27.1	-----
MGH	444	506	0.09
MGH (Inpatient)	112	100	0.78
MGH (Sequenced)	43	34	0.44

Supplemental References

1. RSV-NET interactive dashboard [Internet]. 2022 [cited 2022 Dec 8];Available from: https://www.cdc.gov/rsv/research/rsv-net/dashboard.html

2. Eden J-S, Sikazwe C, Xie R, et al. Off-season RSV epidemics in Australia after easing of COVID-19 restrictions. Nat Commun 2022;13(1):2884.

3. Laham FR, Mansbach JM, Piedra PA, et al. Clinical Profiles of Respiratory Syncytial Virus Subtypes A AND B Among Children Hospitalized with Bronchiolitis. Pediatr Infect Dis J 2017;36(8):808–10.

4. Shi T, McAllister DA, O'Brien KL, et al. Global, regional, and national disease burden estimates of acute lower respiratory infections due to respiratory syncytial virus in young children in 2015: a systematic review and modelling study. Lancet 2017;390(10098):946–58.

5. Hornsleth A, Klug B, Nir M, et al. Severity of respiratory syncytial virus disease related to type and genotype of virus and to cytokine values in nasopharyngeal secretions. Pediatr Infect Dis J 1998;17(12):1114–21.

6. Ruzin A, Pastula ST, Levin-Sparenberg E, et al. Characterization of circulating RSV strains among subjects in the OUTSMART-RSV surveillance program during the 2016-17 winter viral season in the United States. PLoS One 2018;13(7):e0200319.

7. Guide for considering influenza testing when influenza viruses are circulating in the community [Internet]. 2021 [cited 2023 Jan 10];Available from: https://www.cdc.gov/flu/professionals/diagnosis/consider-influenza-testing.htm

8. Hanson KE, Azar MM, Banerjee R, et al. Molecular Testing for Acute Respiratory Tract Infections: Clinical and Diagnostic Recommendations From the IDSA's Diagnostics Committee. Clin Infect Dis 2020;71(10):2744–51.

9. RSV National Trends [Internet]. 2022 [cited 2022 Dec 14];Available from: https://www.cdc.gov/surveillance/nrevss/rsv/natl-trend.html

10. United States Census Bureau [Internet]. [cited 2022 Dec 14];Available from: https://www.census.gov/quickfacts/fact/table/US/PST045221

11. Sanghavi SK, Bullotta A, Husain S, Rinaldo CR. Clinical evaluation of multiplex real-time PCR panels for rapid detection of respiratory viral infections. J Med Virol 2012;84(1):162–9.

12. Matranga CB, Andersen KG, Winnicki S, et al. Enhanced methods for unbiased deep sequencing of Lassa and Ebola RNA viruses from clinical and biological samples. Genome Biol 2014;15(11):519.

13. Park DJ, Dudas G, Wohl S, et al. Ebola Virus Epidemiology, Transmission, and Evolution during Seven Months in Sierra Leone. Cell 2015;161(7):1516–26.

14. Sagulenko P, Puller V, Neher RA. TreeTime: Maximum-likelihood phylodynamic analysis. Virus Evol 2018;4(1):vex042.

15. Huddleston J, Hadfield J, Sibley TR, et al. Augur: a bioinformatics toolkit for phylogenetic analyses of human pathogens. J Open Source Softw [Internet] 2021;6(57). Available from: http://dx.doi.org/10.21105/joss.02906

16. Hadfield J, Megill C, Bell SM, et al. Nextstrain: real-time tracking of pathogen evolution. Bioinformatics 2018;34(23):4121–3.

17. Dudas G. baltic: baltic - backronymed adaptable lightweight tree import code for molecular phylogeny manipulation, analysis and visualisation. Development is back on the evogytis/baltic branch (i.e. here) [Internet]. Github; [cited 2022 Dec 9]. Available from: https://github.com/evogytis/baltic

18. Hunter. Matplotlib: A 2D Graphics Environment. 2007;9:90–5.

19. Suchard MA, Lemey P, Baele G, Ayres DL, Drummond AJ, Rambaut A. Bayesian phylogenetic and phylodynamic data integration using BEAST 1.10. Virus Evol 2018;4(1):vey016.

20. Hasegawa M, Kishino H, Yano T. Dating of the human-ape splitting by a molecular clock of mitochondrial DNA. J Mol Evol 1985;22(2):160–74.

21. Yang Z. Maximum likelihood phylogenetic estimation from DNA sequences with variable rates over sites: approximate methods. J Mol Evol 1994;39(3):306–14.

22. Drummond AJ, Rambaut A, Shapiro B, Pybus OG. Bayesian coalescent inference of past population dynamics from molecular sequences. Mol Biol Evol 2005;22(5):1185–92.

23. Rambaut A, Drummond AJ, Xie D, Baele G, Suchard MA. Posterior Summarization in Bayesian Phylogenetics Using Tracer 1.7. Syst Biol 2018;67(5):901–4.

24. Drummond AJ, Rambaut A. BEAST: Bayesian evolutionary analysis by sampling trees. BMC Evol Biol 2007;7:214.

25. Rambaut A, Lam T, Carvalho L, Pybus O. Exploring the temporal structure of heterochronous sequences using TempEst (formerly Path-O-Gen). Virus Evolution 2016;2(1).

26. Wood DE, Lu J, Langmead B. Improved metagenomic analysis with Kraken 2. Genome Biol 2019;20(1):257.

27. Lemieux JE, Siddle KJ, Shaw BM, et al. Phylogenetic analysis of SARS-CoV-2 in Boston highlights the impact of superspreading events. Science [Internet] 2021;371(6529). Available from: http://dx.doi.org/10.1126/science.abe3261
